# Brain Function and Upper Limb Deficit in Stroke With Motor Execution and Imagery: A Cross-Sectional Functional Magnetic Resonance Imaging Study

**DOI:** 10.3389/fnins.2022.806406

**Published:** 2022-05-19

**Authors:** Zhen-Zhen Ma, Jia-Jia Wu, Xu-Yun Hua, Mou-Xiong Zheng, Xiang-Xin Xing, Jie Ma, Si-Si Li, Chun-Lei Shan, Jian-Guang Xu

**Affiliations:** ^1^Department of Rehabilitation Medicine, Longhua Hospital, Shanghai University of Traditional Chinese Medicine, Shanghai, China; ^2^Center of Rehabilitation Medicine, Yueyang Hospital of Integrated Traditional Chinese and Western Medicine, Shanghai University of Traditional Chinese Medicine, Shanghai, China; ^3^Department of Trauma and Orthopedics, Yueyang Hospital of Integrated Traditional Chinese and Western Medicine, Shanghai University of Traditional Chinese Medicine, Shanghai, China; ^4^School of Rehabilitation Science, Shanghai University of Traditional Chinese Medicine, Shanghai, China

**Keywords:** motor imagery, motor execution, stroke, fMRI, KVIQ

## Abstract

**Background:**

Motor imagery training might be helpful in stroke rehabilitation. This study explored if a specific modulation of movement-related regions is related to motor imagery (MI) ability.

**Methods:**

Twenty-three patients with subcortical stroke and 21 age-matched controls were recruited. They were subjectively screened using the Kinesthetic and Visual Imagery Questionnaire (KVIQ). They then underwent functional magnetic resonance imaging (fMRI) while performing three repetitions of different motor tasks (motor execution and MI). Two separate runs were acquired [motor execution tasks (ME and rest) and motor imagery (MI and rest)] in a block design. For the different tasks, analyses of cerebral activation and the correlation of motor/imagery task-related activity and KVIQ scores were performed.

**Results:**

During unaffected hand (UH) active grasp movement, we observed decreased activations in the contralateral precentral gyrus (PreCG), contralateral postcentral gyrus (PoCG) [*p* < 0.05, family wise error (FWE) corrected] and a positive correlation with the ability of FMA-UE (PreCG: *r* = 0.46, *p* = 0.028; PoCG: *r* = 0.44, *p* = 0.040). During active grasp of the affected hand (AH), decreased activation in the contralateral PoCG was observed (*p* < 0.05, FWE corrected). MI of the UH induced significant activations of the contralateral superior frontal gyrus, opercular region of the inferior frontal gyrus, and ipsilateral ACC and deactivation in the ipsilateral supplementary motor area (*p* < 0.05, AlphaSim correction). Ipsilateral anterior cingulate cortex (ACC) activity negatively correlated with MI ability (*r* = =–0.49, *p* = 0.022). Moreover, we found significant activation of the contralesional middle frontal gyrus (MFG) during MI of the AH.

**Conclusion:**

Our results proved the dominant effects of MI dysfunction that exist in stroke during the processing of motor execution. In the motor execution task, the enhancement of the contralateral PreCG and PoCG contributed to reversing the motor dysfunction, while in the MI task, inhibition of the contralateral ACC can increase the impaired KVIQ ability. The bimodal balance recovery model can explain our results well. Recognizing neural mechanisms is critical to helping us formulate precise strategies when intervening with electrical or magnetic stimulation.

## Introduction

Motor imagery (MI, the mental representation of an action without engaging in its actual execution) is a therapeutically relevant technique to promote motor recovery in neurologic disorders (Cunha et al., 2017; Park et al., 2017). MI shares common neural and psychological bases with physical practice (Horn et al., 2016; Herrador Colmenero et al., 2018). Neurophysiological recordings yield specific changes in cerebral activations during MI similar to those that occur when the action actually occurs (Kato and Kanosue, 2017; Tong et al., 2017). MI can provoke activation of brain areas related to planning, adjustment, automation, and execution of voluntary movements (Vry et al., 2012). Plentiful research has shown that neural processes associated with motor imagery are attributed to the activation of the premotor and parietal areas, primary sensory-motor cortex, and subcortical regions, such as the basal ganglia and cerebellum, as well as corticospinal pathways (Confalonieri et al., 2012; Tong et al., 2017). The therapeutic benefit of MI has been shown in stroke patients with persistent limb motor weakness (Ono et al., 2015; Ushiba and Soekadar, 2016). Thus, combining physical and mental practice has been recommended to enhance upper and lower limb function after stroke and in neurologic rehabilitation (Winstein et al., 2016).

Having a certain level of imaging ability is one of the prerequisites for realizing motor recovery such that stroke patients can benefit from it. The training effect of MI is closely related to MI ability. Theoretically, the more vivid the imagination and the closer to reality, the better the recovery that will be achieved. MI to enhance recovery after subcortical stroke can induce changes in motor-related systems. Factors influencing the motor recovery effects of MI may include the quality of the performance, MI ability, and neuropsychological aspects such as attention and concentration (Sharma et al., 2006).

Essential to evaluating the treatment effects of MI is studying the characteristic changes in MI ability and brain activation pattern in patients with brain injury. Previous studies have found that the MI ability of patients after stroke is reduced, and the brain activation pattern caused by MI is also changed. The disconnections in this network consisting of the prefrontal and parietal regions have been demonstrated to account for the impaired MI ability (McInnes et al., 2016). Oostra et al. (2016) further highlighted the role of the left opercular part of the inferior frontal cortex, basal ganglia, and superior fronto-occipital fasciculus/claustrum in MI.

However, most studies on the effects of MI are limited by their small scale and poor design. Even reviews have reported a high heterogeneity in the methodological quality of the studies and conflicting results (Szameitat, 2012; Herranz-Gómez et al., 2020). There is also limited evidence that people with cortical and subcortical injury have alterations to their motor cortex maps, although this finding has had some conflicting views and more robust fMRI studies are warranted (Ietswaart et al., 2011; Guerra et al., 2017; Herranz-Gómez et al., 2020). How the brain implements MI when the cortical systems involved in motor control are impaired in patients after stroke is still vague and challenged.

Therefore, the purpose of the present study was to assess changes in the neural substrates of MI in patients with stroke, including its association with motor function, and compared with that in non-stroke control participants. To this end, we recruited a group of patients with subcortical stroke and a group of age- and sex-matched healthy participants, employed task-oriented functional magnetic resonance imaging (fMRI) to assess the neural correlates of MI using a task where subjects had to imagine hand gripping, combined with MI scale assessment to more accurately assess MI performance.

## Materials and Methods

### Subjects

This was a single-center, observational, cross-sectional study. The study was conducted at a tertiary grade-A hospital in Shanghai, China, and was approved by their Institutional Review Board and was in accordance with the Declaration of Helsinki (2008). Participants were recruited from the inpatient department and outpatient clinics, and we obtained their informed consent. An abbreviated list of eligibility criteria includes age between 30 and 75 years; at least 1 month from hemorrhagic or ischemic stroke; unilateral hand paresis indicated by a score of 3 or less (full score is 6 points) on upper limb and hand Brunnstrom staging for the motor development stage of the disease; the ability to understand instructions [score above 22 on the mini-mental state examination (MMSE)]. Exclusion criteria consisted of orthopedic restrictions of the upper extremities; botulin toxin injections or other medication influencing the function of the upper extremity; previous history of other neurological conditions; contraindications to an investigation by magnetic resonance imaging (MRI). Twenty-three patients with subacute and chronic stroke and twenty-one healthy controls were included. Patients had a mean age of 53.65 years and (SD 12.09) and control subjects’ mean age was 43.95 years (SD 17.24). Clinical characteristics of patients and control subjects studied are described in Table 1.

**TABLE 1:** Demographic material of patients and healthy controls.

Baseline characteristics	PA (*n* = 23)	HC (*n* = 21)	*P* value
Gender, *n* %			0.142^a^
Male	17 (73.91)	8 (38.10)	
Female	6 (26.09)	13 (61.90)	
Age: Mean (years) (SD)	53.65 (12.09)	43.95 (17.24)	0.063^b^
Time since stroke: Mean (months) (SD)	5.83 (2.69)	–	
Etiology, *n* %		–	–
Ischemic	20 (86.96)	–	–
Hemorrhagic	3 (13.04)	–	–
Paralysis side, *n* %		–	–
Left	11 (47.83)	–	–
Right	12 (52.17)	–	–
MMSE: Mean (SD)	25.70 (3.04)	–	–
Fugl-Myer-UE	21.87 (14.25)	–	–
VIQ-20: Mean (SD)	65 (12.20)	77.14 (10.18)	<0.001
KIQ-20: Mean (SD)	58.44 (13.98)	76.24 (11.38)	<0.001

*KVIQ, Kinesthetic and Visual Imagery Questionnaire; MMSE, Mini-Mental State Examination; HC, healthy controls; FMA-UE, Fugl-Meyer Assessment of the upper extremity. ^a^Chi-square test. ^b^Two independent sample t-test.*

### Clinical Assessments

Upper extremity motor performances of patients with stroke were evaluated by the upper extremity motor part of the Fugl-Meyer Assessment Upper Extremity (FMA-UE) test before fMRI measurement. The FMA-UE is a test based on the concept of sequential stages of motor return (Woytowicz et al., 2017), including items of reflexes, the synergy of the upper extremities, and hand function. Each item is scored on an ordinal 3-point scale to express a maximum motor score for the affected side, with a total score ranging from 0 (hemiplegia) to 66 (normal) (Sullivan et al., 2011). The motor imagery ability for each subject was evaluated according to a modified version of the Kinesthetic and Visual Imagery Questionnaire (KVIQ) developed by Malouin et al. (2007). It assesses both visual (V) and kinesthetic (K) subscales. The questionnaire has 20 items (10 items in each subscale: visual and kinesthetic) and imagery scores use a five-point scale to rate the clarity of the image (5 = the highest level of imagery; 1 = the lowest level of imagery) to assess the vividness of each dimension of MI (clarity of image/intensity of sensation) (Tabrizi et al., 2013).

### Magnetic Resonance Imaging Acquisition

Images were acquired with a 3.0 Tesla scanner (Siemens AG, MAGNETOM Verio) using an 8-channel head coil. A complete fMRI scan sequentially consists of one session of resting-state, two sessions of block design. For functional imaging of resting state, the following parameters were listed as followed: interleaved scanning order, slice number = 43, TR = 3000 ms, matrix size = 64 × 64, FA = 90°, FOV = 192 mm × 192 mm, voxel size = 3 mm × 3 mm × 3 mm, number of acquisitions = 200. For imaging of task state, the fMRI data were measured with an echo-planar imaging sequence (TR/TE = 3000/35 ms, FOV = 220 mm × 220 mm, 39 axial slices, acquisition matrix = 64 × 64, voxel size = 3 mm × 3 mm × 3 mm, number of acquisitions = 100). High-resolution whole-brain anatomical scans were acquired for all subjects as reference for functional activation maps (3D T1-weighted scan: TR = 1900 ms; TE = 2.93 ms, flip angle = 9°, field of view = 240 mm × 240 mm, acquisition matrix = 256 × 256, sagittal acquisition, spatial resolution = 1 mm × 1 mm × 1 mm, interslice space = 0 mm).

### Functional Magnetic Resonance Imaging Experimental Paradigm

**FIGURE 1:**
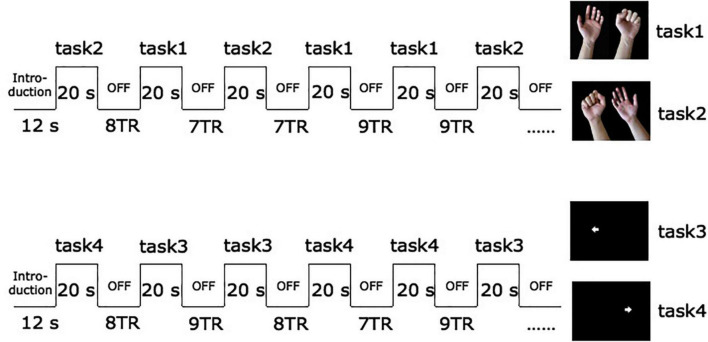
Schematic diagram of fMRI experimental paradigm. The arrow pointing to the left in task 3 prompts subjects to imagine the activity of grasping with the left hand. The arrow towards to the right in task 4 prompts imagery of grasping with the right hand.

Before the fMRI scan, the training subjects actively make fists and relax with their hands, as far as possible in the joint range. The specific method is to lie on the back comfortably with their arms in a supine position supported by a cushion near the subjects’ hips, with the elbows slightly bent to suit themselves, and perform hand movements or imaginary according to the pictures indicated. These two sessions used different visual stimuli. Subjects were required to conduct the execution of regular unilateral grasping and relaxation when the screen presented the target of hand fists and relax picture, and imagine hand gripping exercise when playing the picture of the arrow. During fMRI scanning, participants were asked to perform two different block design paradigms. For the first session (motor execution), the subjects grasped and relaxed the corresponding hands according to the prompts on the screen, with a frequency of 1 Hz. For the second session (motor imagery), the subjects imagined corresponding hand movements according to the arrows (Bajaj et al., 2015; Wang et al., 2016). Each block lasts for 20 s, and a hundred volumes were acquired per session. The sequence of left or right-hand occurrence is pseudo-random, with intervals of 7–9 scans of blank screen pseudo-randomly to avoid the subjects’ expectations of the task. All subjects performed two sessions of four tasks. During the scanning procedure, the researchers in the scanner room performed a visual inspection to confirm that all patients completed the tasks as required (see Figure 1).

### Data Preprocessing

All spatial preprocessing and analysis were performed using SPM12 (Wellcome Trust Centre for Neuroimaging, London, United Kingdom)^1^ on the MATLAB 2014a platform. To ensure the consistency of sides among patients and establishment of the normalization parameters, the brain images of patients with right-sided lesions were flipped over the mid-sagittal plane so that the affected hemisphere corresponded to the left side of the brain for all patients. Preprocessing steps before statistical analysis included slice time correction, motion correction, and spatial normalization to a standard template in MNI space (using the T1 SPM template and resulting in voxels of 3 mm × 3 mm × 3 mm). Normalized images were smoothed with a 6 mm full-width at a half-maximum isotropic Gaussian kernel.

### Data Analysis

Task conditions [motor execution (ME) with unaffected hand (UH) and affected hand (AH), and motor imagery (MI) with UH and AH] were modeled using the standard hemodynamic response function. Block-designed response amplitudes of fMRI data were estimated using the general linear models (GLMs). To correct for signal changes caused by head movement, the six realignment parameters were included in the design matrix. A temporal high-pass filter (cutoff, 128 s) was applied, and temporal autocorrelation was modeled as an AR(1) process (Johnstone et al., 2006). First-level models of individual participant images included each of the session type regressors and six motion parameters to produce estimates for the contrast of interest (motor execution/motor imagery vs. rest, threshold significance set at *p* < 0.001, uncorrected). One sample *t*-tests were applied to create group maps. Then contrast images were analyzed at the second level in a group random-effects analysis using a two-sample *t*-test. The threshold was set at *p* < 0.05 corrected for family wise error (FWE) at the voxel-level in ME condition and *p* < 0.05 corrected for AlphaSim in MI condition.

Correlation analysis was also performed between activation of different brain regions in response to different tasks and behavioral performance scores. Subject-specific activation of the blood oxygenation level dependent (BOLD) signal change was determined by the beta-values extracted from the MarsBaR (Brett et al., 2002) toolkit for correlation analyses. Pearson correlations were calculated between subject-specific activation in each region and behavioral capacity using SPSS (SPSS Inc., Chicago, IL, United States), which controlled the effect of sex and age variables. The model Beta coefficients were calculated for each region-of-interest (ROI), here refers to the brain areas of the difference between groups.

## Results

### Behavioral Results

**FIGURE 2:**
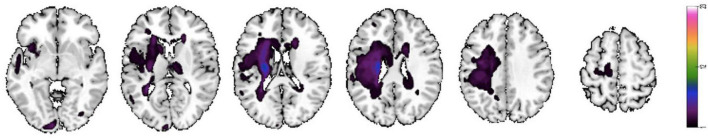
Lesion map of individual stroke lesions. Lesion overlap map of individual lesions in patients with stroke. Maps are overlaid on a T1 template in MNI space. Lesions in the right hemisphere were flipped to the left hemisphere. MNI coordinates of each transverse section (*z*-axis) and a sagittal slice for visualization are given. Color scale indicates the number of patients with a lesion in a given voxel.

All subjects suppressed unexpected movement, and all were compliant during the fMRI task. However, three patients with stroke and one control were excluded because of excessive head motion during motor execution. Two patients with stroke were excluded during the MI task. Twenty patients with stroke (12 left hemisphere strokes; four women) and 20 controls remained in the ME analysis, and 21 patients with stroke remained in the MI analysis (11 left hemisphere strokes; five women). The stroke and control groups were relatively evenly distributed in terms of age and sex (see Table 1 for statistical details). As expected, the groups significantly differed in the Kinesthetic and Visual Imagery Questionnaire (KVIQ) scores, with the stroke group having lower mean scores than those of the control group. The lesion overlap map (see Figure 2) shows a predominance of injuries to the territory of the middle cerebral artery and internal capsule, which may explain the severity of motor deficits in our population.

### Exploratory Analyses

**FIGURE 3:**
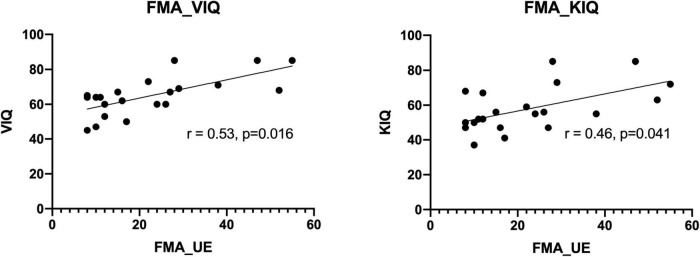
Correlation analyses of KVIQ and FMA-UE scales.

Correlation analyses of MI ability assessed using the KVIQ and motor function assessed using the FMA scale revealed a general consistency between the results. This confirms the close relationship between MI and movement execution. The coefficient related to the Visual Imagery Questionnaire (VIQ) was 0.53 (*p* = 0.016). While the coefficient related to the Kinesthetic Imagery Questionnaire (KIQ) score was 0.46 (*p* = 0.041) (see Figure 3).

### Brain Activation

**FIGURE 4:**
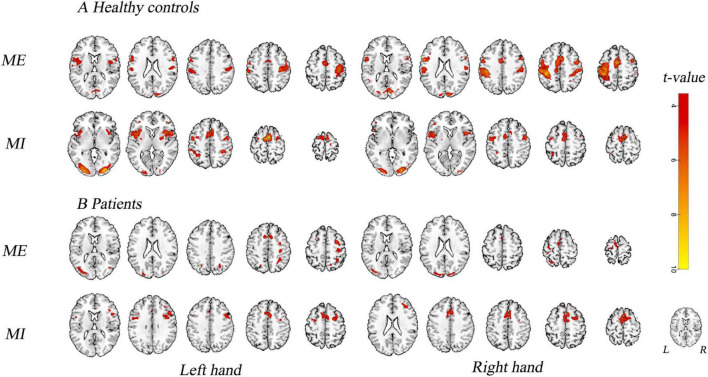
Activation map. Brain activity in different experimental conditions is shown. **(A)** During active motor execution and motor imagery in healthy controls. **(B)** During motor execution and motor imagery in stroke patients. Left hand: activation patterns for tasks performed with the left hand, i.e., unaffected hand (UH) in patients. Right hand: activation patterns for the tasks performed with the right hand, i.e., affected hand (AH) in patients. MNI coordinates of an axial slice for visualization are shown. L, left hemisphere; R, right hemisphere.

Figure 4 and Table 2 show the group activation maps and corresponding Montreal Neurological Institute (MNI) coordinates of the activated brain regions during the conditions in the different tasks performed with the affected hand (AH) and unaffected hand (UH). It is worth noting that during the motor execution task of the AH, the supplementary motor area (SMA) and paracentral lobule, superior parietal gyrus (SPG), and occipital gyrus ipsilateral to the lesion were activated. During motor imagery of the AH, activity was seen in the SMA and SPG in contralateral to the lesion, and medial SPG in the ipsilateral lesion.

**TABLE 2:** Group activation maps and the corresponding MNI coordinates.

Brain regions	Extent	Cluster centroid (MNI)			*t*-value
		*x*	*y*	*z*	
**Healthy controls**					
**Motor execution**					
**Left**					
Precentral_R	1015	30	−18	69	6.7442
Precentral_R	1015	63	6	18	6.0008
Supp_Motor_Area_R	1015	3	−3	63	5.8346
Occipital_Sup_R	136	15	−96	18	6.0568
Cuneus_L	136	−12	−93	15	5.1372
Frontal_Inf_Oper_L	290	−57	9	18	6.0368
Precentral_L	290	−57	6	39	4.7004
Postcentral_L	290	−57	−21	21	4.3101
Parietal_Inf_L	110	−54	−27	48	4.9352
**Right**					
Calcarine_R	245	12	−84	12	10.7458
Cuneus_L	245	−12	−93	15	6.5783
Postcentral_L	2594	−39	−33	54	9.2307
Supp_Motor_Area_R	2594	3	−3	60	8.523
Precentral_R	280	63	9	33	8.5159
Rolandic_Oper_R	280	63	9	9	7.272
Frontal_Mid_2_R	280	42	6	60	6.8944
Temporal_Mid_L	102	−54	−54	0	7.5523
Postcentral_R	270	54	−18	42	7.257
SupraMarginal_R	270	66	−18	24	5.8124
Postcentral_R	270	33	−39	48	5.4606
Precentral_L	2594	−33	−12	60	4.1182
Supp_Motor_Area_R	2594	6	0	63	4.2038
Postcentral_L	2594	−36	−33	60	5.5172
**Motor imagery**					
**Left**					
Occipital_Mid_L	278	−33	−93	−6	9.9191
Calcarine_L	278	−9	−99	−9	5.3696
Occipital_Inf_R	223	33	−90	−6	9.6032
Supp_Motor_Area_R	808	3	3	66	9.5361
Precentral_L	808	−48	−3	54	7.0759
Cingulate_Mid_L	808	−6	6	36	6.155
Frontal_Mid_2_R	61	54	−3	51	6.9917
Frontal_Inf_Oper_R	398	39	15	9	6.8366
Precentral_R	398	60	9	18	5.9663
Rolandic_Oper_L	426	−45	3	9	6.7325
Parietal_Inf_R	48	42	−39	48	5.347
Frontal_Mid_2_L	128	−30	42	12	5.2274
Frontal_Sup_2_R	63	30	3	63	4.9313
Parietal_Inf_L	169	−42	−39	45	4.7775
**Right**					
Frontal_Inf_Oper_R	140	48	15	9	8.9261
Frontal_Inf_Oper_R	140	60	12	27	4.161
Occipital_Inf_R	192	27	−93	−3	7.66
Occipital_Mid_R	192	48	−75	−3	4.3049
Precentral_L	429	−48	−3	42	7.4007
Frontal_Inf_Tri_L	429	−45	15	24	6.2262
Rolandic_Oper_L	429	−60	9	3	5.867
Supp_Motor_Area_L	267	−6	−6	63	6.8058
Supp_Motor_Area_L	267	−6	12	54	5.1171
Precentral_R	79	54	0	48	6.3003
Frontal_Mid_2_R	79	39	0	63	4.3317
Occipital_Mid_L	115	−24	−99	−3	6.0676
Frontal_Mid_2_L	85	−30	42	12	5.6289
Frontal_Inf_Orb_2_L	85	−48	42	−9	5.3301
Postcentral_L	49	−39	−42	63	4.9076
Postcentral_L	49	−30	−39	45	4.3464
**Patients**					
**Motor execution**					
**Left**					
Occipital_Mid_L	76	−33	−87	12	5.2153
Temporal_Mid_L	76	−51	−63	6	4.6995
**Right**					
Occipital_Mid_L	83	−33	−87	12	5.1257
Cuneus_L	83	−9	−93	27	4.4459
Cuneus_R	18	12	−96	21	4.3659
Supp_Motor_Area_L	27	−6	−9	72	4.2314
Parietal_Sup_L	10	−21	−60	66	3.4607
Paracentral_Lobule_L	11	−15	−18	75	3.3982
**Motor imagery**					
**Left**					
Supp_Motor_Area_R	104	9	15	48	5.09
Supp_Motor_Area_L	104	−9	3	63	3.8712
Frontal_Inf_Oper_R	123	54	18	6	4.5508
Frontal_Inf_Oper_R	123	60	15	27	4.4937
Precentral_R	123	45	3	33	4.3664
**Right**					
Frontal_Sup_Medial_L	624	3	21	42	6.1639
Supp_Motor_Area_R	624	3	12	60	5.8429
Frontal_Sup_2_R	624	24	3	57	5.0489
Frontal_Sup_2_R	99	30	42	21	5.158

*x, y, z, coordinates of primary peak locations in the MNI space; t value, peak value of the cluster; p < 0.001, uncorrected.*

### Group Differences in Brain Activation in the Block-Design Scan

**FIGURE 5:**
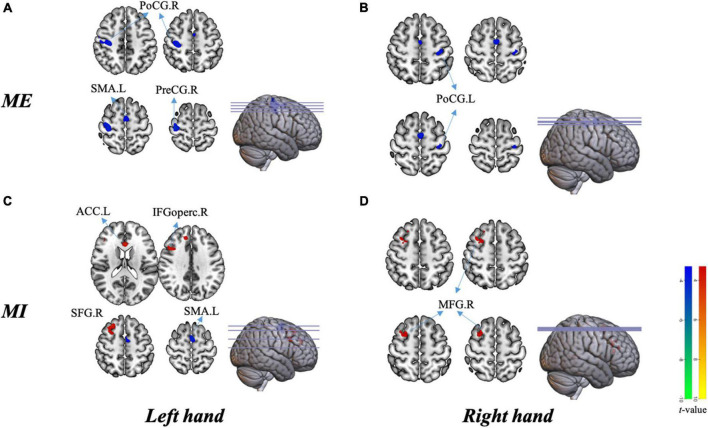
Group differences in brain activation in the block design scan. **(A)** Activation in different areas during motor execution with the left hand [unaffected hand (UH)]. **(B)** Activation in different areas during motor execution with the right hand [affected hand (AH)]. **(C)** Activation in different areas during motor imagery with the left hand (UH). **(D)** Activation in different areas during motor imagery with the right hand (AH). PreCG.R, right precentral gyrus; PoCG.R, right postcentral gyrus; SMA.L, left supplementary motor area; PoCG.L, left postcentral gyrus; ACC.L, left anterior cingulate cortex; SFG.R, right superior frontal gyrus; IFGoperc.R, right opercular region of the inferior frontal gyrus; and MFG.R right middle frontal gyrus.

Figure 5 shows the differenced in whole-brain activation between patients and healthy controls. Table 3 summarizes the corresponding MNI coordinates of the different active brain regions during the different tasks performed with the AH and the UH.

**TABLE 3:** Differences in the brain active regions between PA and HC groups.

Brain regions	Extent	Cluster centroid (MNI)			*t*-value
		*x*	*y*	*z*	
**^a^Left hand_motor execution**					
**HC > PA**					
Precentral_R	215	36	−24	66	4.7355
Postcentral_R	215	48	−21	48	3.6277
Supp_Motor_Area_L	49	−3	−3	57	3.9521
**^a^Right hand_motor execution**					
**HC > PA**					
Postcentral_L	68	−36	−24	51	4.3528
**^b^Left hand_motor imagery**					
**HC > PA**					
Supp_Motor_Area_L	26	−3	−3	63	4.2975
**PA > HC**					
Frontal_Sup_2_R	15	27	27	54	4.1012
Cingulate_Ant_L	10	0	24	18	4.1942
Frontal_Inf_Oper_R	10	45	15	36	3.6699
**^b^Right hand_motor imagery**					
**PA > HC**					
Frontal_Mid_2_R	47	36	12	57	4.1576

*x, y, z, coordinates of primary peak locations in the MNI space; t value, peak value of the cluster. ^a^FWE corrected, P < 0.05. ^b^AlphaSim correction, P < 0.05. The p-value before correction is 0.001.*

#### During Motor Execution

Globally, the UH active grasp movement induced a similar pattern of activation in patients with stroke and healthy controls. However, we observed lower activation in the contralateral precentral gyrus (PreCG), postcentral gyrus (PoCG), and ipsilateral SMA (Figure 5A). During active grasp of the AH, the activation intensity in patients with stroke patients was lower in the contralateral PoCG than in healthy controls (*p* < 0.05, FWE corrected) (Figure 5B).

#### During Motor Imagery

Motor imagery of UH flexion and extension movement induced significant activation of the contralateral superior frontal gyrus (SFG), opercular region of the inferior frontal gyrus (IFGoperc), and ipsilateral anterior cingulate cortex (ACC), with deactivation of the ipsilateral SMA (Figure 5C). Furthermore, we noted significant activation of the contralesional middle frontal gyrus (MFG) during MI of the AH (*p* < 0.05, AlphaSim corrected, Figure 5D).

### Correlation of Clinical Measures and Regions of Interest

**FIGURE 6:**
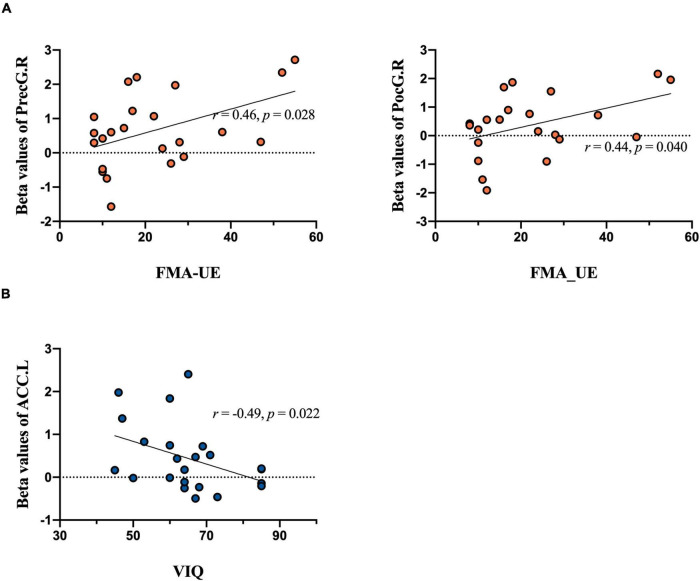
Correlation analysis results. Brain activation during tasks between groups in relation to behavioral performance is included. The upper plane in the figure shows the correlation analysis of extracted beta values from MarsBaR in each region of interest during the motor execution task and the FMA-UE score. **(A)** Significant positive relationship between PreCG.R and FMA-UE score [left panel, unaffected hand (UH)] and PoCG.R and FMA-UE score [right panel, unaffected hand (UH)]. The lower plane in the figure shows the correlation analysis of imagery task-related activity and the VIQ score. **(B)** Significant negative relationship between ACC.L and VIQ score [unaffected hand (UH)].

There were significantly different correlations between behavioral performance and regions of interest across different stimuli. Specifically, the FMA-UE was positively related to activations in the contralateral PreCG (*r* = 0.46, *p* = 0.028), PoCG (*r* = 0.44, *p* = 0.040) in patients during UH active extension movement (Figure 6A). In other words, the motor activation compensation of contralateral motor-related brain area positively correlated with residual motor function. The excitability of the contralateral motor-related brain area positively correlated with behavioral motor performance.

In contrast, MI ability negatively and significantly correlated with activation of the ipsilateral ACC (*r* = –0.49, *P* = 0.022) during the UH imagery task (see Figure 6B). Overall, when the patients do imagery tasks, the activation intensity was inversely proportional to imagery ability. That is, patients require more compensatory activation in the frontal gyrus to perform the mental practice.

## Discussion

Stroke causes different levels of functional impairment, which is often accompanied by widespread activation and connection changes. Researchers are often disappointed by the poor recovery outcomes of various treatment strategies for stroke patients. Current opinions on the central mechanism of the recovery of motor function are believed to depend mainly on reorganization within the sensorimotor cortex but increasing attention is being paid to other cognition-related regions. Recent evidence has suggested that the mirror neuron system (MNS) was involved in motor execution and imagery (Zhang et al., 2018). The classic MNS is understood to be located in the inferior frontal gyrus (IFG), including the ventral premotor cortex (PMv), inferior parietal lobule (IPL), and intraparietal sulcus (IPS) in humans (Rizzolatti and Craighero, 2004; Garrison et al., 2010). Additional brain regions, such as the primary motor cortex, primary somatosensory cortex, and middle frontal cortex are also included in the expanded MNS (Pineda, 2008). However, there have been limited studies that specifically focus on localization of activated brain regions when patients with stroke perform MI. The ambiguity of spatial information has led to a lot of blindly designed stimulation therapies that fail to achieve effective recovery of motor functions. The current study provided a clinical comparative study and assessed cerebral function changes in patients with stroke with upper limb paralysis compared with that in healthy controls during both motor execution and MI tasks. The behavioral correlation analysis was also used to clarify which brain regions are functionally impaired so that we can further get access to the brain recovery mechanisms after stroke.

From the analysis of the motor execution task, we found that there were significant differences in cerebral response in brain regions including the PreCG, PoCG, and SMA. Specifically, regions of the PreCG, as well as the PoCG, significantly positively correlated with FMA-UE scores. Previous studies of motor dysfunction in stroke described major changes in motor-related brain regions, including the primary motor cortex and SMA, and even rewired synaptic connections (Kim et al., 2018; Liu et al., 2020). Our result reached a consistent conclusion with previous studies that exercise execution can mainly be attributed to the activation of motor-related brain regions. However, we surprisingly found that the brain area activation value under the movement of the affected hand did not significantly correlate with the FMA-UE, which implies no obvious change in neural activity in the responsible brain area. We inferred that there are some interactions between bilateral brain regions during the recovery period after stroke, and the movement pattern has changed. The activity of the brain area responsible for the movement of paretic hands may be silent, inhibited, or disordered. Instead, the contralateral brain areas during the UH task positively correlated with FMA-UE, which may be attributed to the excitability of the vicariation regions and a bimodal balance-recovery model that links interhemispheric balancing and functional recovery (Di Pino et al., 2014).

In addition to the motor execution task, we also performed the MI task in a block-design BOLD scan. The results demonstrated altered reactivity within the frontal gyrus, ACC, and SMA. And the ACC has been implicated in the ability to experience MI. The changes in MI ability may explain the pattern of motor regulation and motivational behaviors in motor control (Sharma and Baron, 2013; McInnes et al., 2016). These neural substrates could mediate the generation, maintenance, and manipulation of motor-related images, especially in key processes in visuomotor imagery (Khan et al., 2020). Similarly, we noticed that both tasks demonstrated compensatory neural activation in line with the bimodal balance recovery of the stroke model. This is very similar to the activation mode results displayed in the motion execution task analysis. We found altered activity within related brain regions in the stroke group with lower MI ability. Extending this work would inform the role of MI in the pathophysiology of motor recovery.

Currently, there is a significant body of evidence on the effects of MI (in isolation or combined with physical practice) that share common neural representations with motor execution (Ietswaart et al., 2011; Pan et al., 2019; Khan et al., 2020). In this regard, the neurophysiological equivalence produced between the methods of movement representation and real movement is one of the theories proposed for intervention on functional clinical variables (Munzert et al., 2008; Cuenca-Martínez et al., 2020). Motor impairment and its associated functional activities are regarded as part of a continuum (Langhorne et al., 2009). Motor impairment can be caused by ischemic or hemorrhagic injury to the motor cortex, premotor cortex, motor tracts, or associated pathways in the cerebrum or cerebellum. Motor recovery after stroke is complex and confusing (Langhorne et al., 2009). As found in our motor execution task with the affected limb, the activation of the ipsilateral M1 and SMA showed obvious changes, consistent with previous studies where they played an important role in mediating motor preparation and execution (Pan et al., 2019; Cuenca-Martínez et al., 2020). Effective interventions have demonstrated the ability to improve motor function by re-engaging ipsilesional resources, which appears to be critical and feasible for hand function recovery even in individuals with severe chronic stroke (Wilkins et al., 2020).

The frontal regions are involved in the processing of MI, and they may be differentially responsive (Buch et al., 2012; Yu et al., 2022). The current study adds to this literature that all frontal regions were shown differently between groups while demonstrating greater activation in stroke with the impaired MI ability compared with that of healthy controls. Interestingly, all patients with stroke showed an increased frontal response to the imagery task and a lower M1 and SMA response to the execution task. Together, these findings support the notion that an imaginative load of stroke patients determines the intensity of their frontal lobe activation area. In addition, the worse the imagination ability of patients, the more severe damage to their motor function and even the lower activation of the brain area responsible for the motor output.

The superior frontal gyrus (SFG) has been found to be involved in self-awareness, in coordination with the action of the sensory system (Goldberg et al., 2006). The IFGoperc is cytoarchitecturally known as Brodmann area 44 (BA44) and has been suggested to be involved in music perception (Brown et al., 2006), suppression of response tendencies (Forstmann et al., 2008; Neef et al., 2016), and hand movements (Rizzolatti et al., 2002; Neef et al., 2016). The middle frontal gyrus in our study roughly corresponds with the dorsolateral prefrontal cortex (DLPFC), which is BA46. It plays a central role in executive functions involved in cognitive processes (Cieslik et al., 2013), including working memory, cognitive flexibility (Monsell, 2003), planning (Chan et al., 2008), and regulating self-control. As a review reported, motor imagery primarily recruited a network of bilateral premotor, rostral inferior and middle superior parietal, basal ganglia, and cerebellar regions (Hardwick et al., 2017). There was also a relatively small cluster in the left MFG consistent with the DLPFC (Hardwick et al., 2017). However, the clinical effect of treatment based on MI for stroke is still not ideal and there is notable heterogeneity between studies. Developing a standard protocol for assessing technical and clinical outcomes is required to provide evidence on efficiency and efficacy that need to be developed for future clinical treatments.

In conclusion, these results demonstrate the dominant effects of MI dysfunction that exists in stroke during the processing of motor execution. There is a significant relationship between motor imagery ability and motor function, which highlights the dependence of these two variables. Recognizing neural mechanisms is critical to helping us formulate precise strategies when intervening with electrical or magnetic stimulation. According to our results, the scheme of activating motor-related areas and inhibiting frontal areas during electrical stimulation may be a direction worth considering. Our research has shed light on the mechanism of motor imagery participation in movement and evaluated the correlation between active neural substrates and psychological processes in the KVIQ, which provides new evidence for the role of MI in stroke treatment. Future directions include investigating directional connections within these neural substrates related to imagery processes as well as establishing underlying functional networks for post-stroke patients.

## Limitations

First, the study was a cross-sectional research and had a relatively small sample size. Second, the patient sample included a wide range of stroke types, lesion position, and stroke types. Third, inter-individual variability may contribute to the absence of significant results of the regression analyses on ME and MI involving the AH. Subjective assessments on participation surveys under task conditions cannot be ignored although these questionnaires are regarded as validated tools to estimate MI ability.

## Data Availability Statement

The raw data supporting the conclusions of this article will be made available by the authors, without undue reservation.

## Ethics Statement

The studies involving human participants were reviewed and approved by Medical Ethics Committee of Yueyang Hospital. The patients/participants provided their written informed consent to participate in this study.

## Author Contributions

Z-ZM, J-JW, and J-GX contributed to the conception and design of the study. Z-ZM organized the database and wrote the first draft of the manuscript. X-XX performed the statistical analysis. X-YH, M-XZ, S-SL, and C-LS wrote sections of the manuscript. All authors contributed to manuscript revision, read, and approved the submitted version.

## Conflict of Interest

The authors declare that the research was conducted in the absence of any commercial or financial relationships that could be construed as a potential conflict of interest.

## Publisher’s Note

All claims expressed in this article are solely those of the authors and do not necessarily represent those of their affiliated organizations, or those of the publisher, the editors and the reviewers. Any product that may be evaluated in this article, or claim that may be made by its manufacturer, is not guaranteed or endorsed by the publisher.

## Footnotes

1
https://www.fil.ion.ucl.ac.uk/spm/software/spm12/
